# Proportion of bacterial isolates, their antimicrobial susceptibility profile and factors associated with puerperal sepsis among post-partum/aborted women at a referral Hospital in Bahir Dar, Northwest Ethiopia

**DOI:** 10.1186/s13756-019-0676-2

**Published:** 2020-01-13

**Authors:** Alemale Admas, Baye Gelaw, BelayTessema, Amsalu Worku, Addisu Melese

**Affiliations:** 1 Department of Medical Laboratory Science, College of Medicine and Health Sciences, Bahir Dar University, Bahir Dar, Ethiopia; 2 Department of Medical Microbiology, School of Biomedical and Laboratory Science, College of Medicine and Health Sciences, University of Gondar, Gondar, Ethiopia; 3 Department of Gynecology and Obstetrics, School of Medicine, Bahir Dar University, Bahir Dar, Ethiopia

**Keywords:** Puerperal sepsis, Gram-positive bacteria, Gram-negative bacteria, Antimicrobial susceptibility, Felege Hiwot referral hospital, Women, Ethiopia

## Abstract

**Background:**

Puerperal sepsis is any bacterial infection of the genital tract that occurs after childbirth. It is among the leading causes of maternal morbidity and mortality especially in low-income countries including Ethiopia. The aim of this study was to determine the proportion of bacterial isolates, their antimicrobial susceptibility profile and factors associated with puerperal sepsis among post-partum/aborted women at a Referral Hospital in Bahir Dar, Northwest Ethiopia.

**Methods:**

A cross sectional study was conducted from January to May 2017 among 166 post-partum/aborted women admitted to Felege Hiwot Referral Hospital for medical services and suspected for puerperal sepsis.. Socio-demographic data and associated factors were collected using structured questionnaire. Bacteria were isolated and identified from blood samples on Trypton soya broth, blood, Chocolate and MacConkey agars following standard bacteriological procedures. The VITEK 2 identification and susceptibility testing system was used to determine the antimicrobial susceptibility profiles of bacterial isolates. Data were entered and analyzed using SPSS version 20. Factors associated with puerperal sepsis were considered statistically significant at *P*-value < 0.05.

**Results:**

The overall proportion of bacterial isolates among post-partum/aborted women was 33.7% (56/166); of which 55.4% was caused by Gram-negative and 44.6% was by Gram-positive bacteria. The most frequently isolated bacteria were *Escherichia coli* (32.1%) from Gram-negatives and *Staphylococcus aureus (*33.9%) from Gram-positives. The proportion of other isolates was *(*7.2%) for Coagulase Negative *Staphylococci* (CoNS), (12.5%) for *Klebsiella pneumoniae*, *(*10.7%) for *Acinetobacter baumanni* and *(*3.6%) for *Raoultella ornithinolytica*. All isolates of Gram-positive and Gram-negative bacteria were resistant to tetracycline (100%). The gram negatives show resistance to Cefazolin (72.7%), Tetracycline (93.9%) and Ampicillin (100%). The overall prevalence of multidrug resistance (MDR) was 84%. Women having multiparous parity were more likely to develop puerperal sepsis than primiparous parity (AOR 4.045; 95% CI: 1.479–11.061; *P* < 0.05). Other socio-demographic and clinical factors had no significant association with puerperal sepsis.

**Conclusion:**

About one third of post-partum/aborted women suspected for puerperal sepsis were infected with one or more bacterial isolates. Significant proportion of bacterial isolates showed mono and multi-drug resistance for the commonly prescribed antibiotics. Women with multiparous parity were more likely to develop puerperal sepsis than primiparous parity.

## Background

Puerperal sepsis is any bacterial infection of the genital tract that occurs after child birth usually in the first 24 h [1] or at any time between the rupture of membranes or labor and the 42nd days of postpartum [2]. Following vaginal delivery, approximately 6–7% of women experience puerperal sepsis. Home birth in unhygienic conditions, poor nutrition, prim parity, anemia, prolonged rupture of membranes (PROM), prolonged labor, multiple vaginal examinations, caesarean section, instrumental deliveries, and postpartum hemorrhage are some of the risk factors for puerperal sepsis [3].

World Health Organization (WHO) estimated that about 350,000 maternal deaths occur during labor and childbirth of which 15% were associated with puerperal sepsis [4]. Puerperal sepsis causes at least 75,000 maternal deaths every year and mostly occurs in low-income countries with a distribution of 11.6% in Asia, 9.7% in Africa, 7.7% in Latin America and the Caribbean compared to the 2.1% in developed countries [5, 6]. In Nigeria, puerperal sepsis accounted for 12% of maternal deaths [7] In Ethiopia, puerperal sepsis accounted for about 13% of all maternal deaths and became one of the top four causes of mortality [8].

Antibiotics are among the successful interventions developed to treat bacterial diseases including puerperal sepsis, but bacteria could develop resistance [9]. Resistance to antimicrobial agents has emerged as a public health problem at an alarming rate. Infecting agents capable of developing resistance to multiple drugs (MDR) have enhanced rate of morbidity and mortality [10]. The dynamic nature of bacteria resistant to antimicrobial agents can lead to prolonged illness, increased health care expenditures and risk of death.

Ethiopia has adopted the sustainable development goals (SDGs) to reduce maternal mortality to < 70/100,000 by the year 2030. To achieve this target and significantly reduce maternal mortality, identifying the causes and contributors of puerperal sepsis is paramount. Therefore, this study was aimed to determine the proportion of bacterial isolates, their antimicrobial susceptibility profile and factors associated with puerperal sepsis among post-partum/aborted women suspected for puerperal sepsis at Felege Hiwot Referal Hospital, Northwest Ethiopia.

## Methods

### Study design, area, and period

A cross sectional study was conducted from January to May 2017 among postpartum/aborted women admitted to Felege Hiwot Referral Hospital for medical services and suspected of puerperal sepsis.. The Hospital is found in Bahir Dar; the capital city of the Amhara Regional State. In the city, there were 14 healthcare serviceinstitutions (one government referral hospital, one primary hospital, two general private hospitals and ten health centers) during data collection.

### Study populations

Postpartum/aborted women (any type of abortion) admitted to Felege Hiwot Referral Hospital during the study period. Women were suspected for puerperal sepsis if they develop fever of > 38.5 ^0^c, pelvic pain, abnormal genital discharge with offensive smell and delay in the reduction of the size of uterus to the 42nd day of postpartum/abortion.

### Inclusion and exclusion criteria

Women with sign and symptoms of puerperal sepsis at the time of admission or who developed sepsis during their stay in the hospital during the study period were included in the study. However, women with puerperal sepsis but started antibiotic therapy and those unable to give the interview were excluded.

### Sample size and sampling technique

The sample size was determined using a single population proportion formula: n = z^2^ p (1-p)/ d^2^; where: n = the number of postpartum/aborted women; z = standard normal distribution value at 95% CI (z = 1.96); p = the prevalence of puerperal sepsis. Taking p at 8.4% from study conducted in Addis Ababa, Ethiopia [11]; d = the margin of error, taken as 5%. Accordingly, the sample size was ($\mathrm{n}=\frac{{\left(\mathbf{1}.\mathbf{96}\right)}^{\mathbf{2}}\mathbf{0.084}\left(\mathbf{1}-\mathbf{0.084}\right)}{{\mathbf{0.05}}^{\mathbf{2}}}$) estimated at 119. Additional 47 women who fulfilled the inclusion and exclusion criteria were admitted to the hospital during the study period and included in the study. Hence, the final total sample size was 166. Convenient sampling was used to select the study participants.

### Data collection

#### Socio-demographic and clinical data

Socio-demographic characteristics of the study participants was collected using a pretested and structured questionnaire by BSc nursing graduates. The questionnaire was prepared in English and translated to the local Amharic language. The questionnaire was pretested on a sample of 5% of study participants at University of Gondar specialized Hospital and amended accordingly. The questionnaire was prepared based on the WHO education material on puerperal sepsis and customized to this study [1]. The clinical data of each woman was retrieved from their medical records.

#### Nutritional status assessment

The nutritional status of each woman was measured by mid upper arm circumference (MUAC using tape meter. MUAC less than 23 cm were considered as under nutrition [12].

#### Assessment of anemia

Postpartum/aborted women were screened for anemia using automated hematology analyzer (Cell-DYN 1800, Abbott diagnostic, USA). Each blood sample was investigated for total hemoglobin (Hgb) concentration. The Hgb thresholds were used to classify anemia. Anemia was categorized as mild (Hgb level 10 to 10.9 g/dl), moderate (Hgb level 7.0 to 9.9 g/dl) and severe (Hgb level < 7.0 g/dl) per WHO criteria [13].

#### HIV screening

The HIV status of each woman was collected from the medical record of each patient. Screening for HIV in the hospital was done with the First Response (Premier Medical Corporation, India), Unigold (Trinity Biotech Manufacturing, Ireland) and Vikia (bioMerieux SA, France) test algorithm.

#### Blood collection and processing

Blood was collected from each woman to detect the presence and frequency of bacteria.. Two bottles of blood (5 ml for each bottle) was collected aseptically from two different sites of peripheral veins. Blood was inoculated directly into Trypton soya broth (Oxoid Ltd. Basingstoke, Hampshire, UK) and incubated aerobically at 37 °C. The growth of visible colonies were observed daily for the first 3 days. Subcultures were made on blood agar, Chocolate agar, and MacConkey agar (Oxoid Ltd. Basingstoke, Hampshire, UK) and examined for growth after 24–48 h of incubation. The procedure was repeated and negative blood cultures were reported after the 7th day of incubation. Blood cultures positive for bacterial growth were further analyzed for colony isolation. Bacterial isolates were identified by gram stain and VITEK 2 identification and susceptibility testing system (bioMerieux, Inc., Hazelwood, USA). Standard biochemical tests were also used as backup identification modalities [14].

#### Antimicrobial susceptibility testing

Antimicrobial susceptibility tests were done for each identified bacteria using the VITEK 2 identification and susceptibility testing system. The VITEK 2is an automated, walkway system that works on the principle of photometry. Briefly, pure colony of the isolated bacteria was emulsified in 2 ml of 0.85% normal saline. Bacterial suspensions with an optical density of 0.5–0.63 was taken as acceptable bacterial concentration. Reagents were then immersed to the suspension and transferred to VITEK 2 system for antimicrobial susceptibility testing [15].

#### Quality control

Training for data collectors and supervisors about the study and data collection procedures was given. Consistency and completeness of data was also checked on daily bases. The working standards of materials, equipment, culture media and procedures were strictly followed. The sterility of culture media was ensured by incubating 5% of each batch at 37 °C for 24 h. The performance of each media was checked by inoculating standard strains of *E. coli* (ATCC 25922) for Gram-negative bacteria, *S. aureus* (ATCC 25923) for Gram-positive bacteria and *N. gonorrhoeae* (ATCC49226) for fastidious bacteria [16]. The VITEK 2 system was also verified using known strains.

#### Data processing and analysis

Socio-demographic and laboratory data were entered and analyzed using SPSS version 20. Descriptive statistics was presented as frequencies and percentages in tables and figures. Logistic regressions were used to assess factors associated with puerperal sepsis. Odds ratio with its 95% CIs was used to assess the presence of association and its significances was declared at *P*-values < 0.05.

## Results

### Socio-demographic and clinical characteristics

During the study period, a total of 756 women were admitted for postpartum/abortion medical services. Of these, 166 women suspected for puerperal sepsis and enrolled in the study. The mean age of the study participants was 27.3 years (SD ± 6.2). Data on educational status showed that 42.2% were unable to read and write, 36.7% had secondary school and above while 21.1% attended only elementary school. Majority (134, 80.7%) of cases of puerperal sepsis was related with labor while 32 (19.3%) was related with abortion. Of the laboring mothers, 91 (67.9%) gave birth through caesarean section (CS) and 43 (32.1%) through spontaneous vaginal delivery (SVD). Half (83, 50%) of the study participants were living in rural areas and the remaining were urban residents (Table 1). Twenty (12%) of women did not follow antenatal care. Among the 134 laboring mothers, eleven women (6.6%) were found to be HIV positive.

**Table 1:** Socio demographic characteristics of women suspected of puerperal sepsis at Felege Hiwot Referral Hospital, Bahir Dar, Northwest Ethiopia, 2017, (*n* = 166)

Variable		Frequency N, (%)	Bacterial isolates	
			Yes, *N* (%)	No, *N* (%)
Age	18–24	68 (41)	22 (32.4)	46 (67.6)
	25–34	69 (41.6)	17 (24.6)	52 (75.4)
	35–42	29 (17.5)	17 (58.6)	12 (41.4)
Educational Status	Illiterate	70 (42.2)	29 (41.4)	41 (58.6)
	1–4 grade	14 (8.4)	8 (57.1)	6 (42.9)
	5–8 Grade	21 (12.7)	6 (28.6)	15 (71.4)
	Secondary and Above	61 (36.7)	13 (21.3)	38 (78.7)
Occupation	Government Employee	36 (27.7)	7 (19.4)	29 (80.6)
	Farmer	65 (39.2)	26 (40)	39 (60)
	Merchant	17 (10.2)	8 (47)	9 (53)
	House Wife	37 (22.2)	13 (35.1)	24 (64.9)
	Other	11 (6.7)	2 (18.2)	9 (81.8)
Residence	Rural	86 (51.8)	26 (30.2)	60 (69.8)
	Urban	80 (48.2)	30 (37.5)	50 (62.5)
Religion	Orthodox	148 (89.2)	48 (32.4)	100 (67.6)
	Muslim	18 (10.8	8 (44.4)	10 (55.6)

### Proportion of bacterial isolates

**Fig. 1:**
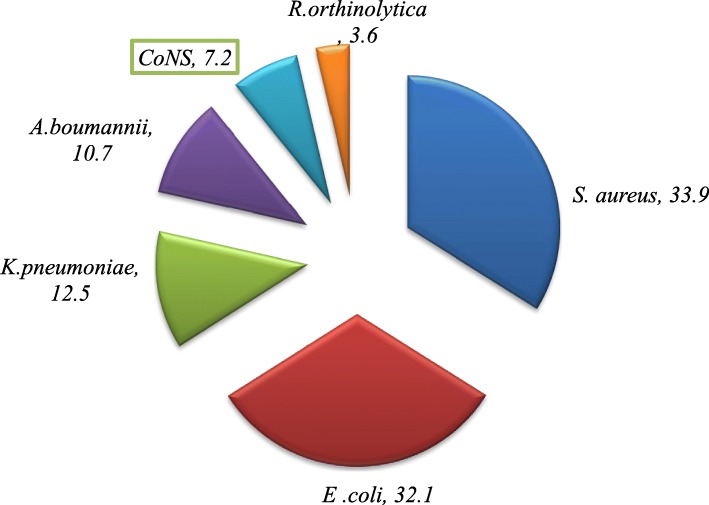
Frequency (%) of bacterial isolates from women suspected of puerperal sepsis at Felege Hiwot Referral hospital, Bahir Dar, Northwest Ethiopia, 2017

The overall prevalence of bacterial isolates among women suspected of puerperal sepsis was 33.7% (56/166). The proportion of Gram-negative bacteria was 58.9% (33/56) and that of Gram-positive was 41.1% (23/56). Overall, *S. aureus* was the dominant isolate (33.9%; 19/56) followed by *E. coli* (32.1%; 18/56) (Fig. 1). The proportion of Gram-negative isolates was 54.5% (18/33) for *E. coli,* 21.2% (7/33) for *K. pneumonaie* 18.2% (6/33) for *A. baumannii* and that of *Raoultella orinthinolytica was* 3.6% (2/33). On the other hand, the proportion of Gram-positive isolates was 82.6% (19/23) for *S. aureus* and 17.4% (; 4/23) for CoNS.

### Antimicrobial susceptibility profiles of gram-positive bacteria

Gram-positive bacteria demonstrated high rate of resistance to Tetracycline (100%), Erythromycin (87%) and Penicillin (61%). However, gram positive bacteria showed low rate of resistance to Ciprofloxacin (4.3%), Vancomycin (13%) and Ceftriaxone (13%). *S. aureus* isolates were susceptible to Vancomycin (100%) but resistant to Cefoxitin (47.4%), Erythromycin (84.2%) and Tetracycline (100%). The drug susceptibility profile of CoNS isolates showed resistance to Penicillin (100%), Ampicillin (100%), Tetracycline (100%) and Erythromycin (100%). None of the Gram-positive bacteria were susceptible for all antimicrobials tested (Table 2).

**Table 2:** Antimicrobial susceptibility profile of Gram-positive bacteria isolated from women suspected of puerperal sepsis at Felege Hiwot Referral Hospital, Bahir Dar, Northwest Ethiopia, 2017

Bacterial Isolates	Resistance Rate, *N* (%)										
	P	AMP	VAN	CXT	SXT	CIP	CRO	TE	CAF	E	DA
*S.aureus* (*n* = 19)	10 (52.6)	8 (42.1)	0	9 (47.4)	6 (31.6)	2 (10.5)	2 (10.5)	19 (100)	7 (36.8)	16 (84.2)	5 (26.3)
CoNs (*n* = 4)	4 (100)	4 (100)	3 (75)	3 (75)	2 (50)	1 (25)	2 (50)	4 (100)	1 (25)	4 (100)	3 (75)
Total (*n* = 23)	14 (61)	12 (52)	3 (13)	12 (52)	8 (35)	3 (13)	4 (17)	23 (100)	8 (35)	20 (87)	8 (35)

*CoNS* Coagulase negative *Staphylococcus*, *DA* Clindamycin, *CXT* Cefoxitin, *E* Erythromycin, *P* Penicillin, *SXT* Trimethoprim-sulfamethoxazole, *TE* Tetracycline, *AMP* Ampicillin, *CRO* Ceftriaxone, *CAF* Chloramphenicol, *CIP* Ciprofloxacin

### Antimicrobial susceptibility profiles of gram-negative bacteria

Gram-negative bacteria showed high rate of sensitivity to Gentamicin (78.8%) and Ciprofloxacin (75.8%) while high rate of resistance was observed for Cefazolin (72.7%), Tetracycline (93.9%) and Ampicillin (100%). Species specific resistance of Gram-negative bacteria showed that *E. coli* were resistant to Ampicillin (100%) and Tetracycline (100%). Nevertheless, all isolates of *E* .*coli* were susceptible to Ciprofloxacin and Gentamicin. *K. pneumoniae* isolates were highly resistant to Ampicillin (100%) andTetracycline (100%). However; relatively sensitive isolates were observed for Trimethoprim/Sulfamethoxazole (57.1%), Ceftriaxone (57.1%) and Gentamicin (57.1%). *A. boumanni* isolates were resistant to trimethoprim/Sulfamethoxazole (100%) and Ampicillin (100%). Isolates of *Raoultella orinthinolytica* were resistant for most antibiotics tested except to Tetracycline (Table 3).

**Table 3:** Antimicrobial susceptibility profile of Gram-negative bacteria isolated from women suspected of puerperal sepsis at Felege Hiwot Referral Hospital, Bahir Dar town, Northwest Ethiopia, 2017

Bacterial isolates	Resistance rate, *N* (%)									
	SXT	AMC	TE	CIP	GEN	CRO	CZN	AMP	CAZ	CAF
*E.coli* (*n* = 18)	6 (33.3)	7 (38.8)	18 (100)	0	0	7 (38.8)	10 (55.5)	1 8 (100)	6 (33.3)	2 (11.1)
*K.pneumoniae* (*n* = 7)	3 (42.8)	4 (57.1)	7 (100)	4 (57.1)	3 (42.8)	4 (57.1)	7 (100)	7 (100)	4 (57.1)	3 (42.8)
*A.baumannii* (*n* = 6)	6 (100)	5 (83.3)	6 (100)	2 (33.3)	2 (33.3)	3 (50)	5 (83.3)	6 (100)	3 (50)	2 (33.3)
*R.orinthinolytica* (*n* = 2)	1 (50)	2 (100)	0	2 (100)	2 (100)	2 (100)	2 (100)	2 (100)	2 (100)	2 (100)
Total = 33	16 (48.5)	18 (54.5)	31 (93.9)	8 (24.2)	7 (21.2)	16 (48.5))	24 (72.7)	33 (100)	15 (45.5)	9 (27.2)

*SXT* Trimethoprim-Sulfamethoxazole, *AMC* Amoxicillin-clavulanate, *TE* Tetracycline, *Amp* Ampicillin, *CIP* Ciprofloxacin, *GEN* Gentamicin, *CRO* Ceftriaxone, *CAZ* Ceftazidam and *CAF* Chloramphenicol

### The proportion of multidrug resistant (MDR) bacterial isolates

Among the 56 isolates tested for susceptibility, 84% showed resistance to 3 or more classes of antibiotics. The proportion of bacteria resistant to 3 different antimicrobials was 21.4% while 55.4% were resistant to more than three classes of antibiotics. The proportion of MDR *S. aureus* was 52.6% (10/19). The study also showed high rate of MDR CoNS (Table 4).

**Table 4:** Multi-drug resistance profile of bacterial isolates from women suspected of puerperal sepsis at Felege Hiwot Referral Hospital, Northwest Ethiopia, 2017

Bacterial pathogens	Patterns of drug resistance								Total MDR (%)
	R0 (%)	R1 (%)	R2 (%)	R3 (%)	R4 (%)	R5 (%)	R6 (%)	R7 (%)	
*S. aureus (n = 19)*	0	3 (15.8)	6 (31.6)	1 (5.3)	1 (5.3)	1 (5.3)	1 (5.3)	6 (31.6)	10 (52.6)
*CoNS (n = 4)*	0	0	0	0	1 (25)	0	0	3 (75)	4 (100)
*E. coli (n = 18)*	0	0	0	8 (44.4)	3 (16.6)	0	1 (5.5)	6 (33.4)	18 (100)
*K.pnuemonaie (n = 7)*	0	0	0	3 (42.9)	0	0	0	4 (57.1)	7 (100)
*A.baumannii (n = 6)*	0	0	0	0	1 (16.6)	1 (16.6)	0	4 (66.7)	6 (100)
*R.orinthinolytica (n = 2)*	0	0	0	0	0	0	0	2 (100)	2 (100)
*Total (n = 56)*	0	3 (5.4)	6 (10.7)	12 (21.4)	5 (8.9)	2 (3.6)	2 (3.6)	22 (39.3)	47 (84)

Key: R_0_: susceptible to all antimicrobials; R1–7: resistance to 1, 2, 3, 4, 5, 6 & 7 antimicrobials

**Fig. 2:**
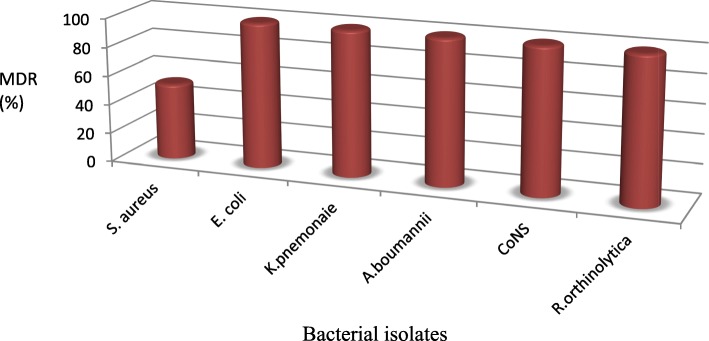
The proportion of multidrug resistant bacterial isolates from women suspected of puerperal sepsis at Felege Hiwot Referral Hospital, Bahir Dar, Northwest Ethiopia, 2017

The antimicrobial susceptibility profile of Gram-negative isolates showed significant rate of MDR. The proportion of MDR *E. coli* was 44.4% and that of *K. pnemonaie* was 42.9%. In addition, 66.7% of *A. baumanii* and all *R. ornithinolytica* isolates were MDR (Fig. 2).

### Factors associated with puerperal sepsis

In this study; parity, HIV status, antenatal care, duration of labour, membrane rupture, mode of delivery, diabetes mellitus, method of placenta removal, hypertension, anemia and place of delivery were evaluated as possible factors associated with puerperal sepsis. In the binary analysis; multiparous parity, antenatal care, diabetes mellitus and anemia showed s association with puerperal sepsis (*P* < 0.05). The multivariable regression analysis showed that only being multiparous parity had statistically significant association with puerperal sepsis. Women that had multiparous parity were four times more likely to develop puerperal sepsis compared to primiparous women (AOR = 4.045; 95% CI: 1.479–11.061; *P* = 0.006). Other socio-demographic and clinical variables had no significant association with puerperal sepsis (Table 5).

**Table 5:** Bivariate and multivariable analysis of factors associated with of puerperal sepsis at Felege Hiwot Referral Hospital, Bahir Dar, Northwest Ethiopia, 2017

Variable		Bacterial isolate					
		No	Yes	COR (95% CI)	*P* –Value	AOR (95%CI)	*P*-value
Parity	Primiparous	60	20	1	0.023^a^	1	0.006^a^
	Multiparous	50	36	2.160 (1.113–4.192)		4.045 (1.479–11.061)	
HIV	No	104	51	1	0.399		
	Yes	6	5	1.699 (0.495–5.833)			
ANC	Booked	92	54	1	0.030^a^	1	
	Un booked	18	2	0.189 (0.042–0,848)		0.000	
Duration of labor	< 18 h	59	29	1	0.094	1	0.101
	> = 18 h	24	22	1.865 (0.899–3.868)		2.058 (0.868–4.879)	
Membrane rupture	0–8 h	59	29	1	0.094	1	0.761
	> 8 h	24	22	1.865 (0.899–3.868)		1.151 (0.464–2.859)	
Mode of delivery	Vaginal	28	15	1	0.603		
	CS	55	36	1.220 (0.574–2.599)			
Placenta removal	Spontaneous	19	3	1	0.703		
	Cord Traction	8	0	0.000			
	Manual	1	2	2.923 (0.239–35.68)			
Diabetes mellitus	No	107	49	1		1	0.117
	Yes	3	7	5.095 (1.264–20.54)	0.022^a^	3.471 (0.733–16.433)	
Hypertension	No	104	55	1	0.291		
	Yes	6	1	0.315 (0.037–2.684)			
Anemia	None	50	33	1	0.040^a^	1	0.36
	Mild	23	4	0.264 (0.084–0.832)		0.111 (0.020–0.608)	
	Moderate	20	15	1.136 (0.510–2.531)		0.846 (0.287–2.494)	
	Sever	17	4	0.357 (0.110–1.154)		0.229 (0.045–1.155)	
Place of delivery	Heath institute	81	47	1		1	0.729
	Home	29	9	0.535 (0.233–1.226)	0.139	0.816 (0.179–3.707)	

^a^ Significant at *p* < 0.05

## Discussion

Puerperal sepsis is one of the leading causes of preventable maternal morbidity and mortality [17]. In the present study, about one third of women suspected of puerperal sepsis were infected with one or more bacterial isolates with an overall proportion of 33.7%. Previous study from Ethiopia reported similar findings (33.3%) [18]. On the other hand, the proportion of bacterial isolates causing puerperal sepsis was lower than reports from Nigeria (82.7%), Sudan (72.9%), and Bangladesh (84%) [16, 19, 20]. These discrepancies might be attributable to cultural, health seeking behavior, health care coverage, period of studies conducted and other socioeconomic factors.

In this study, the proportion of bacterial isolates causing puerperal sepsis was higher than reports from Nepal (19.7%) and Uganda (17%) [21, 22]. This difference can be explained by fact that two-third (67.9%) of women gave birth through caesarean section (CS). Postnatal infections (pyrexia, endometritis, and puerperal sepsis), thrombosis and excessive blood loss are indeed higher after caesarean section [23].

The prevalence of Gram-positive bacteria causing puerperal sepsis was 41.1% and *S. aureus* was the dominant isolate (33.9%). This is consistent with previous study that report *S. aureus* as one of the major bacteria isolated from puerperal septic patients [16, 19]. *S. aureus* is frequently found on the skin and can be easily acquired during delivery [24].

The prevalence of Gram-negative bacteria was 58.9% and *E coli* was the dominant isolate (32.1%). This finding is higher than reports *isolated* from women with puerperal sepsis in Bangladesh (14.5%) [20] and Nigeria (15.8%) [25]. It is known that *E. coli* are among the commonest bacterial isolates causing blood stream infections, wounds and other complications in humans [26, 27]. K*. pnemonaie* with a prevalence of (12.5%) was found to be the third frequently isolated bacteria among women with puerperal sepsis in our study. It is recently emerged as a significant cause of hospital acquired infections (urinary tract infection, pneumonia and septicemia) especially among immune compromised individuals [28, 29] which can be sources of infection for puerperal sepsis.

Two very uncommon bacterial pathogens namely *A. baumannii* and *R. orinthinolytica* were isolated from the blood culture of women suspected of puerperal sepsis. *A. baumannii* is an opportunistic bacteria affecting people with compromised immune systems and increasingly important hospital acquired infection [30]. It has been noted for its apparent ability to survive on artificial surfaces for extended period of time that can allow it to persist in the hospital environment as a source of puerperal sepsis [31].

This study revealed that *E. coli* isolates were susceptible to Ciprofloxacin (100%) and Gentamicin (100%). Similar susceptibility profiles were reported form Ethiopia around Jimma and Gondar [32, 33]. On the other hand, resistance to Ampicillin (100%) was observed among *E. coli* isolates consistent with reports from India and Ethiopia [20, 32]. This is because; *E. coli* has resistant genes for beta-lactam agents including ampicillin [34–36] and has selective pressure excreted by overuse of the antibiotics.

Multiple drug resistance (MDR) is an emerging public health threat that has been reported in different countries [37]. In our study, the overall proportion of MDR isolates was 84%. Similar finding were reported from Uganda (80%) that majority of puerperal sepsis were caused by MDR bacteria [22]. *S. aureus* were highly resistant to Tetracycline (consistent with other studies) [30] and unusually to Erythromycin. Similarly, 47.4% of *S. aureus* isolates showed resistance to Cefoxtin which is relatively lower than reports from India (53.2%) [38, 39]. More than half (52.6%) of *S. aureus* isolates were resistant to three or more tested antimicrobials (MDR) This can be explained by its unique features to adapt antimicrobial pressures and genetic competence to acquire antibiotic resistant genes from other strains.

Relatively high proportion of MDR was observed among *E .coli, A. baumannii,* and *K. pneumonaie* isolates. *K. pneumonaie* has chromosomal and plasmid encoded beta-lactam hydrolyzing enzymes [40]. *A. baumannii* is clinically important pathogen and causes septicemia with widespread resistance to various antibiotics [41]. There are reports that showed resistance of *R. ornithinolytica* to ampicillin and other commonly used antibiotics which can be associated with the presence of β-lactamases [42, 43].

Puerperal sepsis can be associated with different socio-demographic and clinical factors. In the present study, statistically significant association was observed between multiparous parity and puerperal sepsis. Women that had multiparous parity were four times more likely to develop puerperal sepsis compared to primiparous women (AOR = 4.045; 95% CI: 1.479–11.061; *P* = 0.006). Similar finding was reported from Pakistan that high prevalence of puerperal sepsis was observed among women of multiparous parity (78.29%) [44].

Although it is not significantly associated, women with age greater than 34 years had high proportion of culture positive bacterial infection (58.6%). This is supported by previous study from Pakistan indicating that majority (65.11%) of women admitted with puerperal sepsis were above 30 years of age [44]. Previous study reported that rural residence, educational level, family monthly income, prolonged labour (> 24 h), delivery by caesarean section, ANC follow up, ruptured membrane (> 24 h before delivery), frequent vaginal examination and being referred were reported as significant factors associated with puerperal sepsis [45]. But in our study HIV status, antenatal care, duration of labour, membrane rupture, mode of delivery, diabetes mellitus, method of placenta removal, hypertension, anemia and place of delivery had significant association with puerperal sepsis.

### Limitation of the study

The limitation of the study include: investigation for anaerobic bacteria that can cause significant puerperal sepsis was not done. The study was conducted only in a public referral hospital that lacks representativeness and difficult to generate generalized conclusions. Convenient sampling was used to select study participants that can allow missing of cases. In addition, the nutritional status of post-partum/aborted women was measured by MUAC that may not be enough to determine nutritional status as a single measurement.

## Conclusion

About one third of post-partum/aborted women suspected for puerperal sepsis were infected with one or more bacterial isolates. *E. coli* and *S. aureus* were the most dominant bacteria isolated from post-partum/aborted women. Significant proportion of bacterial isolates showed mono and multi-drug resistance rates. Relatively high rate of resistance was observed to Tetracycline and Ampicillin. The very uncommon bacterial isolates of *A. baumanni* and *R. ornithinolytica* showed high rate of resistance to the commonly prescribed antibiotics. Post-partum/aborted women with multiparous parity were more likely to develop puerperal sepsis than primiparous parity. Systematic surveillance for the commonly prescribed antibiotics and uncommon bacterial isolates is recommended to maintain the emergence and spread of antimicrobial resistance. In addition; awareness about post-partum/abortion complications and available cares are needed.

## Data Availability

Data were collected from puerperal sepsis suspected women admitted to Felege Hiwot Referral Hospital, Bahir Dar, Northwest Ethiopia. The datasets are available from the corresponding author upon reasonable request through alemalea@gmail.com but will not be shared publicly to ensure patient confidentiality.

## Abbreviations

AIDS: Acquired Immune Deficiency Syndrome
ATCC: American Type Cell Culture
CLSI: Clinical Laboratory Standard Institute
CoNS: Coagulase Negative Staphylococcus
CS: Cesarean Section
DM: Diabetic Mellitus
HIV: Human Immune Deficiency Virus
MDR: Multi Drug Resistance
MUAC: Mid Upper Arm Circumference
PROM: Prolonged rupture of membranes
SDGs: Sustainable Developmental Goals
SVD: Spontaneous vaginal delivery
TMP/SMX: Trimethoprim/Sulphametaxone
VRE: Vancomycin Resistance *Enterococci*
WHO: World Health Organization
